# Intensive support recommendations for critically-ill patients with suspected or confirmed COVID-19 infection

**DOI:** 10.31744/einstein_journal/2020AE5793

**Published:** 2020-05-29

**Authors:** Thiago Domingos Corrêa, Gustavo Faissol Janot de Matos, Bruno de Arruda Bravim, Ricardo Luiz Cordioli, Alejandra del Pilar Gallardo Garrido, Murillo Santucci Cesar de Assuncao, Carmen Silvia Valente Barbas, Karina Tavares Timenetsky, Roseny dos Reis Rodrigues, Hélio Penna Guimarães, Roberto Rabello, Frederico Polito Lomar, Farah Christina de La Cruz Scarin, Carla Luciana Batista, Adriano José Pereira, João Carlos de Campos Guerra, Bárbara Vieira Carneiro, Ricardo Kenji Nawa, Rodrigo Martins Brandão, Antônio Eduardo Pereira Pesaro, Moacyr Silva, Fabricio Rodrigues Torres de Carvalho, Cilene Saghabi de Medeiros Silva, Ana Claudia Ferraz de Almeida, Marcelo Franken, Marcele Liliane Pesavento, Raquel Afonso Caserta Eid, Leonardo José Rolim Ferraz

**Affiliations:** 1 Hospital Israelita Albert Einstein São PauloSP Brazil Hospital Israelita Albert Einstein , São Paulo , SP , Brazil .

**Keywords:** Coronavirus, COVID-19, Respiratory insufficiency, Respiratory distress syndrome, adult, Intensive care units

## Abstract

In December 2019, a series of patients with severe pneumonia were identified in Wuhan, Hubei province, China, who progressed to severe acute respiratory syndrome and acute respiratory distress syndrome. Subsequently, COVID-19 was attributed to a new betacoronavirus, the severe acute respiratory syndrome coronavirus 2 (SARS-CoV-2). Approximately 20% of patients diagnosed as COVID-19 develop severe forms of the disease, including acute hypoxemic respiratory failure, severe acute respiratory syndrome, acute respiratory distress syndrome and acute renal failure and require intensive care. There is no randomized controlled clinical trial addressing potential therapies for patients with confirmed COVID-19 infection at the time of publishing these treatment recommendations. Therefore, these recommendations are based predominantly on the opinion of experts (level C of recommendation).

## INTRODUCTION

In December 2019, in Wuhan, province of Hubei, China, many patients developed severe pneumonia and presented severe acute respiratory syndrome (SARS) and acute respiratory distress syndrome (ARDS). ^( 1 , 2 )^ Later, the disease disseminated to other regions of China and to many countries in different continents, characterizing a pandemic. ^( 3 )^ The disease was attributed to a new betacoronavirus, named severe acute respiratory syndrome coronavirus 2 (SARS-CoV-2). ^( 4 )^ The disease caused by this virus was recently named coronavirus disease 2019 (COVID-19). ^( 5 )^

Patients with COVID-19 present mostly with fever, cough, dyspnea, myalgia, and fatigue. ^( 6 )^ Although most have a favorable progression, ^( 2 )^ approximately 20% develop severe forms of the disease, including acute hypoxemic respiratory failure (AHRF), SARS, ARDS and acute kidney failure (AKF) requiring admission to the intensive care unit (ICU). ^( 1 , 2 , 7 )^ It has also been found that some groups, especially the elderly and those with other underlying diseases, have greater risk of developing multiple organ dysfunction (MODS) and, ultimately, dying. ^( 2 , 8 )^

As of the publication of these treatment recommendations, no randomized controlled clinical trial (RCT) had yet evaluated potential treatments for patients with confirmed COVID-19 infection. ^( 5 , 9 )^ A search on ClinicalTrials.gov ^( 10 )^ using the term “COVID -19” resulted in 179 RCTs in the recruitment phase. A detailed narrative review of the specific treatments used to treat COVID-19 has been published recently. ^( 9 )^

Therefore, the material provided in these recommendations is based mostly on experts’ opinion. Consequently, they should be considered with caution by healthcare professionals, considering the recommendation level C (case report, including cohort studies or lower quality case-control studies). ^( 11 )^

## CRITERIA FOR ADMISSION TO THE INTENSIVE CARE UNIT

At least one of the following criteria is necessary for admission to the ICU:

- Patients who need supplemental oxygen (nasal cannula – NC O _2_ >3.0L/minute) to maintain peripheral oxygen saturation (SpO _2_ ) >94% or respiratory rate (RR) ≤24rpm.
- Patients who require non-invasive ventilation (NIV) to maintain SpO _2_ >94% or RR ≤24rpm.
- Acute respiratory failure requiring invasive mechanical ventilation (MV).
- Patients with hemodynamic instability or shock, defined as hypotension (systolic blood pressure - SBP <90mmHg or mean arterial pressure - MAP <65mmHg), or signs of poor organic or peripheral perfusion (changes in consciousness levels, oliguria, lactate ≥36mg/dL, among others), using or not vasopressors.
- Sepsis with hypotension, need for vasopressor or lactate ≥36mg/dL.
- Septic shock according to the Sepsis-3 criteria. ^( 12 )^

## VENTILATORY SUPPORT

Non-invasive ventilation and high-flow nasal cannula:

- The use of NIV in positive airway pressure modalities at two pressure levels (BiPAP) and high flow nasal cannula (HFNC) is contraindicated because of the large production of aerosol.
- A short NIV test (30 minutes) can be conducted for patients with AHRF. The NIV test should be done with maximum parameters of inspired oxygen fraction (FiO _2_ ) ≤50%, or positive pressure (PP) with delta ≤10cmH _2_ O and expiratory positive airway pressure (EPAP) ≤10cmH _2_ O ( Figure 1 ).
  **Figure 1:**
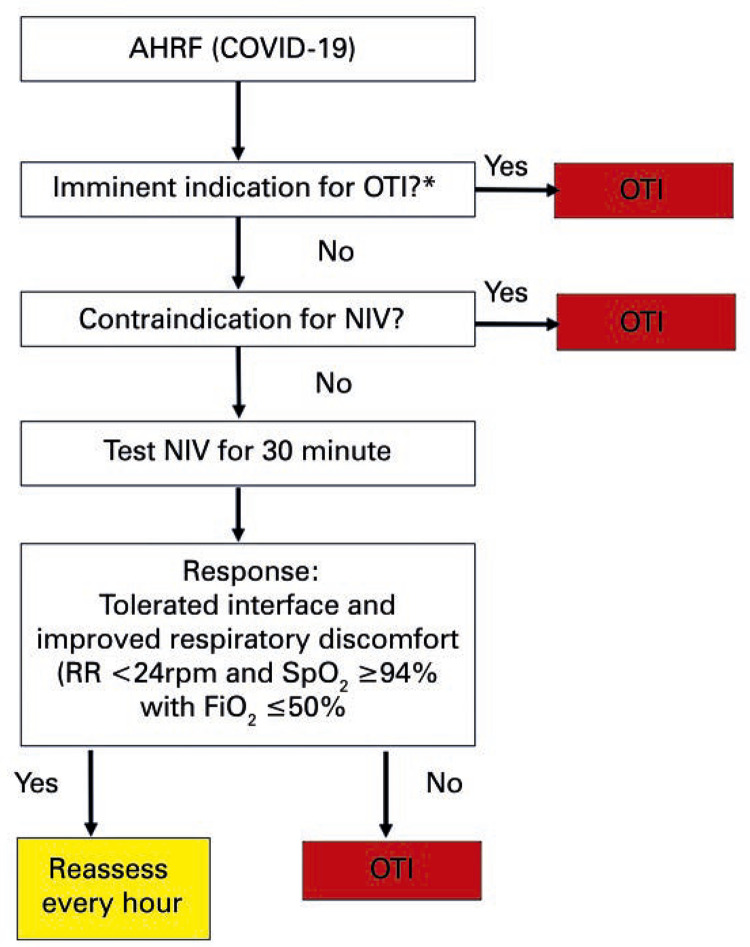
Recommendations for the use of non-invasive ventilation in patients with suspected or confirmed COVID-19 infection * The 30-minute NIV test is carried out with a non-vented mask (without exhalation valve) and a double circuit in a mechanical ventilator with barrier filter at the expiratory outlet. AHRF: acute hypoxemic respiratory failure; OTI: orotracheal intubation; NIV: noninvasive ventilation; RR: respiratory rate; SpO _2_ : peripheral oxygen saturation; FiO _2_ : inspired oxygen fraction.
- To enable safe use of the NIV interface, a non-vented orofacial mask (without exhalation valve) coupled to a dual circuit, specific for MV and connected to it is recommended.
- Use barrier filter at the expiratory outlet of the mechanical ventilators.
-  NIV success criteria: patient tolerates interface and improves respiratory distress (RR <24rpm and SpO _2_ ≥94% with FiO _2_ ≤50%) after 30 minutes of NIV.
- Definition of NIV dependence: need to remain in NIV greater than or equal to 4 hours over a 6-hour period to maintain RR <24rpm and SpO2 ≥94%.
- NIV-dependent patients must be transferred to the ICU early, for immediate orotracheal intubation (OTI).

## CRITERIA FOR OROTRACHEAL INTUBATION

-  Patients who need oxygen supplementation through nasal cannula NO _2_ C >5L/minute or NIV with FiO _2_ >50% or delta PP >10cmH _2_ O or EPAP >10cmH _2_ O to maintain SpO _2_ >94% or RR ≤24rpm.
-  Patients who do not adapt to or do not tolerate the NIV interface.
-  NIV-dependent patients.

## PROCEDURES FOR OROTRACHEAL INTUBATION

- Team apparel during the OTI procedure: waterproof apron, sterile gloves, N95 mask, hair cap, goggles and face shield.
- Only professionals who will actively participate in the procedure remain in the room. We recommend a trained professional stays by the door of the room for possible support during the OTI.
- Prepare all materials for OTI, including capnograph, drugs, fluids and vasopressors, before beginning the procedure and out of room.
- Vasopressors (norepinephrine) and crystalloids must be prepared and kept ready for infusion before beginning the procedure, due to the potential risk of hypotension after OTI.
- Use orotracheal cannula with subglottic suction and closed suction system in all patients undergoing OTI and MV.
- All OTI procedures must be performed with direct videolaryngoscopy.
- All patients must be intubated with rapid sequence intubation (RSI).
- SpO _2_ drop below 70% is a common event immediately after OTI. Therefore, pre-oxygenation (100% O _2_ ) and the proper preparation of both patient and materials to be used for OTI are crucial.
- Avoid ventilation with bag-valve-mask before OTI, due to the increased production of aerosol.
- In patients not using NIV at the time of OTI: pre-oxygenate with reservoir mask at the lowest possible air flow, to maintain effective oxygenation. Avoid assisted ventilation with the bag-valve-mask or use of supraglottic devices, due to the potential aerosol production and team contamination.
- In patients using NIV at the time of OTI: start RSI with the patient in NIV. Remove NIV only to intubate, *i.e* ., do not remove the mask before the OTI.
- Sequence of medications for SRI:
  - Fentanyl 50 to 100mcg, intravenous (IV).
  - Etomidate 0.3mg/kg IV or propofol 2mg/kg IV, at least 2 minutes after fentanyl infusion.
  - Lidocaine 2% without vasoconstrictor 40mg IV.
  - Neuromuscular blockade with succinylcholine 1.0mg/kg IV (or rocuronium 1.2mg/kg IV, if succinylcholine is contraindicated), to facilitate OTI and avoid cough during the procedure.
- After checking the adequate position of the orotracheal tube with the capnograph and installing the heat and moisture exchanger (HME) filter between the tube and the capnograph, the patient may be connect to the ventilator, installing the high efficiency particulate arrestance (HEPA) filter at the expiratory outlet of the ventilator to the environment.

## INITIAL ADJUSTMENTS OF THE MECHANICAL VENTILATOR

The following initial MV parameters are recommended immediately after OTI:

-  Pressure-controlled volume (PCV).
-  Tidal volume (VT) of 6mL/kg of predicted weight. ^( 13 )^
-  Initial positive end-expiratory pressure (PEEP) of 15cmH _2_ O.
-  Respiratory rate between 20 and 24rpm, to maintain minute ventilation (VE) between 7 and 10L/minute.
-  Driving pressure (plateau pressure minus PEEP) ≤15cmH _2_ O.
-  Initial SpO _2_ targeting between 92 and 96%.
-  Initial end-tidal carbon dioxide (EtCO2) targeting between 30 and 45.
-  Measuring arterial blood gases 1 hour after OTI is suggested for possible adjustments to the initial MV parameters.

## PROTECTIVE MECHANICAL VENTILATION STRATEGY

**Figure 2:**
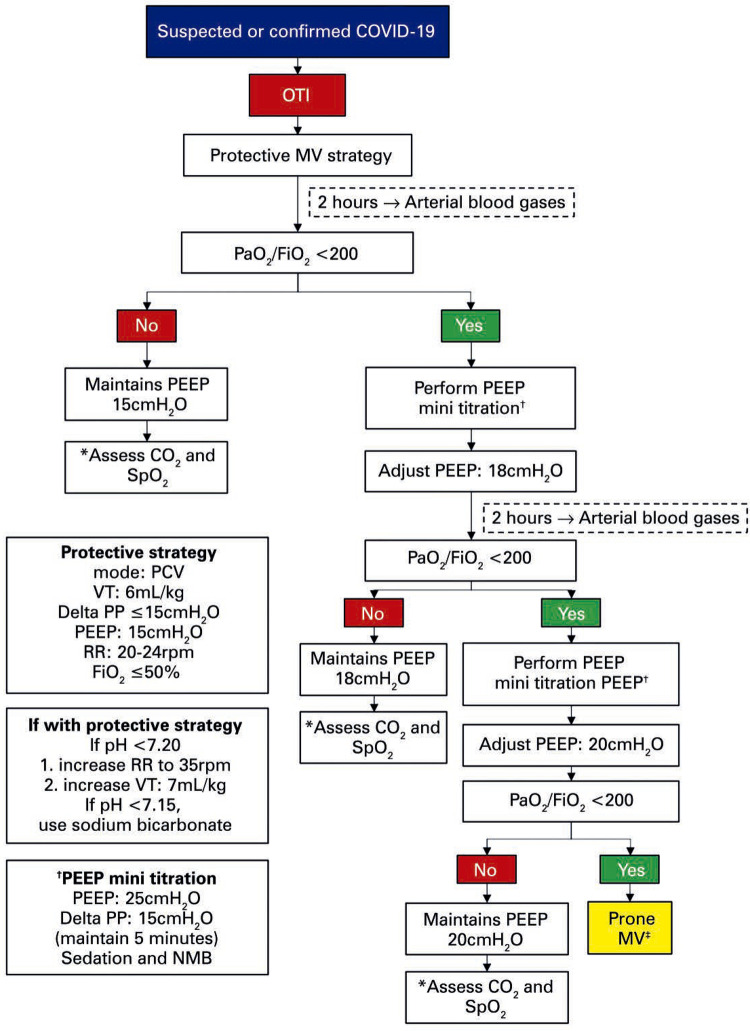
Flow chart for mechanical ventilation in patients with suspected or confirmed diagnosis of COVID-19 * If increase in partial pressure of arterial CO _2_ and pH <7.20, evaluate the possibility of reducing PEEP by 2cmH _2_ O and reassess; ^†^ PEEP mini titration with the PEEP parameters 25cmH _2_ O and delta PP 15cmH _2_ O for 5 minutes. Optimize sedation and NMB; ^‡^ follow prone MV protocol. If no response, perform PEEP mini titration again. Adjust PEEP to 18cmH _2_ O. OTI: orotracheal intubation; MV: mechanical ventilation; PaO _2_ /FiO _2_ : partial arterial oxygen pressure and inspired oxygen fraction; PEEP: positive end-expiratory pressure; CO _2_ : carbon dioxide; SpO _2_ : peripheral oxygen saturation; prone MV: mechanical ventilation in prone position; PCV: pressure-controlled volume; VT: tidal volume; RR: respiratory rate; PP: positive pressure; NMB: neuromuscular blocker.

The MV strategy applied for patients with suspected or confirmed diagnosis of COVID-19 is displayed in figure 2 .

## CRITERIA TO BEGIN WEANING FROM VENTILATION

- After 24 hours stable with PEEP initially adjusted according to figure 2 , try reducing PEEP by every 1cmH _2_ O, every 8 hours, for partial pressure of arterial oxygen to fraction of inspired oxygen ratio (PaO _2_ /FiO _2_ ) >300.
- If the patient is chronically hypoxemic, use PaO _2_ /FiO _2_ >250 to reduce PEEP.
- Modify the pressure-volume control to spontaneous only when PEEP ≤15cmH _2_ O, FiO _2_ <50%, and Richmond agitation-sedation scale (RASS) >-5. ^( 14 )^

## CRITERIA TO BEGIN SPONTANEOUS BREATHING TRIAL

**Figure 3:**
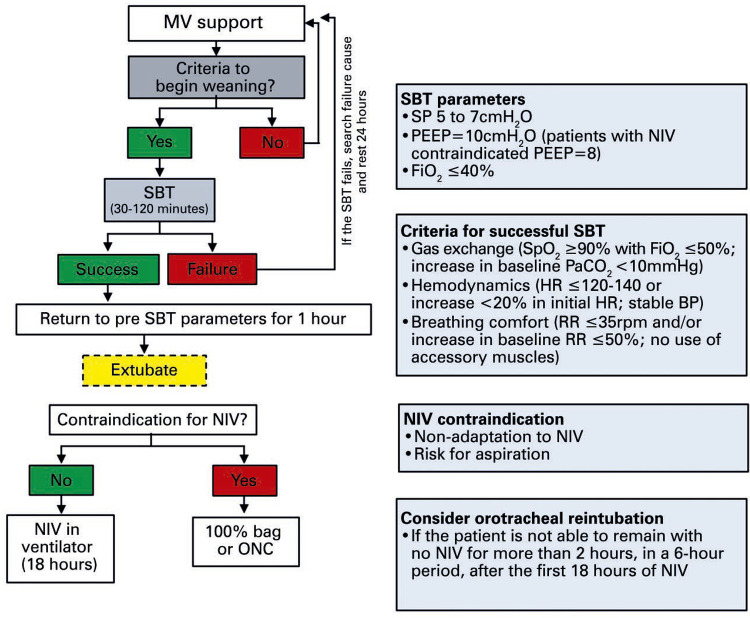
Flowchart for weaning from mechanical ventilation in patients with suspected or confirmed diagnosis of COVID-19 MV: mechanical ventilation; SBT: spontaneous breathing trial; NIV: noninvasive ventilation; ONC: oxygen nasal cannula; 100% bag: oxygen reservoir mask (non-rebreather); SP: support pressure; PEEP: positive end-expiratory pressure; FiO _2_ : fractional inspired oxygen; SpO _2_ : peripheral oxygen saturation; PaCO _2_ : partial pressure of carbon dioxide; HR: heart rate; BP: blood pressure; RR: respiratory rate.

**Figure 4:**
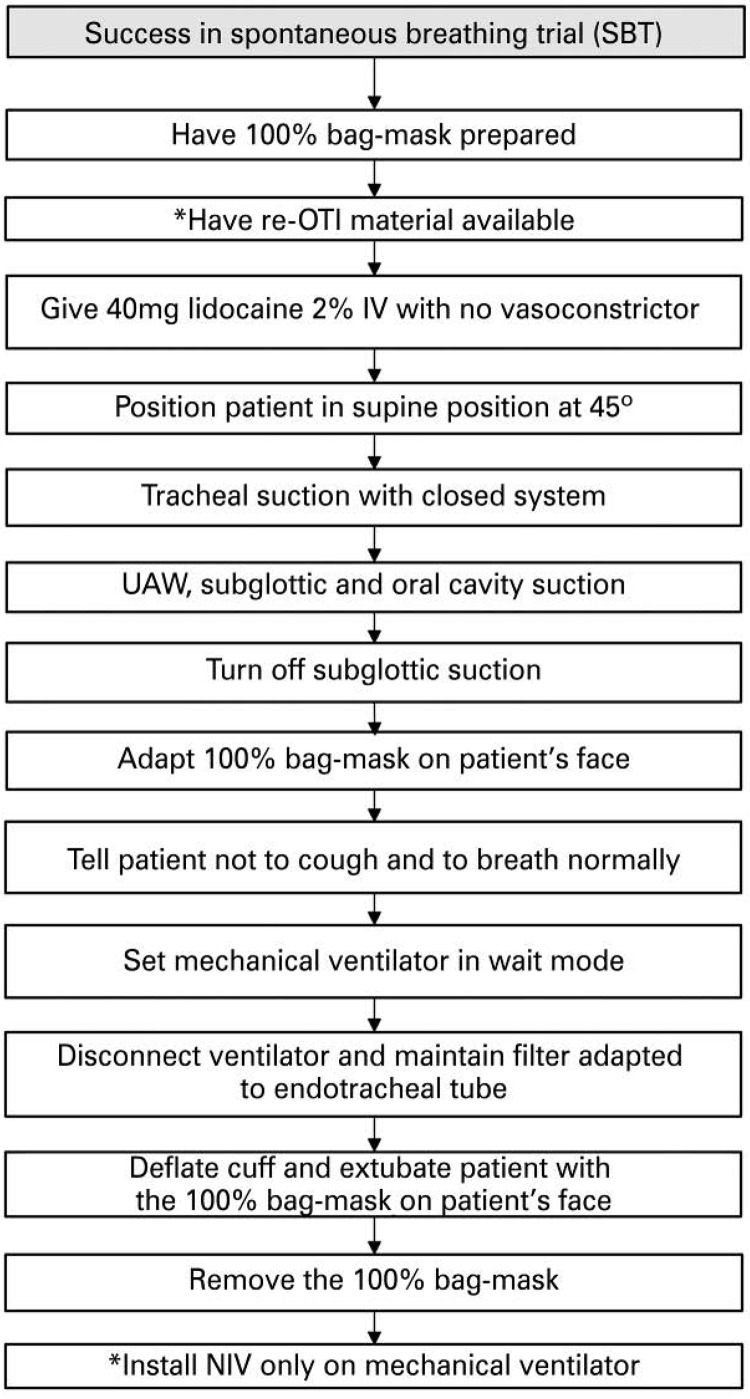
Flowchart of care after spontaneous breathing trial and extubation of patients with suspected or confirmed diagnosis of COVID-19 * If patient presents contraindication to NIV, keep in 100% bag-mask and check often. Keep NIV for 18 hours after extubation. After this period, if the patient is unable to stay 2 hours with no NIV, Re-OTI. SBT: spontaneous breathing trial; OTI: orotracheal intubation; IV: intravenous route; UAW: upper airway; 100% bag-mask: oxygen reservoir mask (non-rebreather); NIV: non-invasive ventilation.

To conduct the spontaneous breathing trial (SBT) we recommend the patient should (figures 3 and 4):

- Remain for 24 hours in pressure support ventilation (PSV) with PEEP=10cmH _2_ O, FiO _2_ <40% and support pressure (SP) ≤10cmH _2_ O, and maintain airway occlusion pressure for the first 100 milliseconds (P0.1) ≤4cmH _2_ O.
- Present adequate level of consciousness: RASS 0-2 ^( 14 )^ or close to baseline.
- Be hemodynamically stable, defined by no need for vasopressors or <0.2mcg/kg/minute norepinephrine or dobutamine stable dose or weaning, and appropriate tissue perfusion marker levels.
- After 24 hours with the MV parameters as described above and adequate gas exchange, characterized by pH 7.3 with arterial pressure of carbon dioxide (PaCO _2_ ) <55mmHg; PaO _2_ ≥60mmHg with FiO _2_ ≤40%; PEEP ≤10cmH _2_ O; PaO _2_ /FiO _2_ ≥250; SpO _2_ ≥90%, start SBT (figures 3 4).
- Do not perform cuff leak test ^( 15 )^ before extubating patients due to the risk of aerosol production.

## ANALGESIA AND SEDATION

- The association between propofol (maximum dose of 3.0mg/kg/hour) IV and fentanyl (25 to 50mcg/hour; maximum dose 100mcg/hour) IV is the first choice for sedation and analgesia in patients on MV due to COVID-19.
- If the propofol dose is greater than 3mg/kg/hour, it should be combined with midazolam, at a dose of 0.02 to 0.2mg/kg/hour, IV.
- Recommended sedation targeting during the first 48 hours of MV: RASS -5.
- Recommended sedation targeting after the first 48 hours of MV:
  - PaO _2_ /FiO _2_ >250 and PEEP ≤15cmH _2_ O and FiO _2_ ≤50%: RASS -3 to zero.
  - PaO _2_ /FiO _2_ ≤250 and/or PEEP ≥15cmH _2_ O: RASS -4 to -5.
- If required, additional doses to control agitation during MV should be given:
  - Propofol: 10 to 40mg IV bolus.
  - Midazolam: 3 to 5mg IV bolus.
  - For additional pain control (analgesia) during MV, additional IV bolus of fentanyl should be given (50mcg IV) and association with regular analgesics ( *e.g* . dipyrone or paracetamol) to spare using opioids.
- Regarding the use of neuromuscular blockers, (NMB), administering cisatracurium 0.15mg/kg IV bolus is recommended, followed by infusion of 1 to 4mcg/kg/minute in continuous infusion pump (CIP), when one of the following is observed:
  - Persistent severe asynchrony characterized by worsening of oxygenation and ventilation after adjusting MV and sedation, in patients with PaO _2_ /FiO _2_ between 150 and 200;
  - PaO _2_ /FiO _2_ <150 with PEEP >15cmH _2_ O.
- In patients on NMB, based on current guidelines, ^( 16 , 17 )^ we recommend:
  - Bi-spectral index (BIS) targeting 40 to 60, with burst suppression rate (SR) > zero.
  - Train of four (TOF): targeting zero (assess once a day, if possible).
  - When BIS monitoring is not possible, deep sedation should be given (RASS-5) before beginning NMB infusion.
- We suggest maintaining NMB for 48 hours after starting it for COVID-19 patients. Patients on NMB for more than 48 hours, if PaO _2_ /FiO _2_ >200 and PEEP <20cmH _2_ O, stop NMB and reassess in 24 hours.
- For patients in continuous sedation, daily revision of the possibility of decreasing sedation is recommended if pH >7.30 and:
  - If PaO _2_ /FiO _2_ >200, try replacing midazolam with propofol, if midazolam is being used.
  - If PaO _2_ /FiO _2_ >300, try replacing propofol with dexmedetomidine (0.3 to 1.3mcg/kg/hour).
  - Concurrently try to gradually reduce the dose of fentanyl.
- During the attempt to decrease sedation, if the patient presents asynchrony with drop in SpO _2_ and/or hemodynamic instability, we suggest:
  - Administer 10 to 40mg propofol IV bolus and 25 to 50mcg fentanyl IV bolus.
  - Administer one dose of neuroleptics (Quetiapine 25 to 50mg via nasoenteral tube – NET – or, if not possible, use haloperidol IV).
  - If there is no improvement after 20 minutes, deepen the sedation, decreasing by -2 in the RASS, or as far as RASS-5, and reevaluate within 24 hours. If two or more boluses are needed in a 6-hour period, consider deepening sedation and reassessing within 24 hours.
  - After 48-hour sedation, in patients chronically using psychoactive drugs, reconcile before considering the reduction of continuous sedation.

## GENERAL MEDICAL SUPPORT

- Hand hygiene always. Install contact and aerosol precautions during the entire ICU stay.
- Avoid drugs that prolong the QT interval (example: zofran, bromopride, fluconazole, metoclopramide, antiarrhythmic drugs), especially if using macrolides or chloroquine.
- Do not provide inhalation therapy, so as to avoid aerosol production. Use bronchodilators with spacer in case of bronchospasm.

## ANTIBIOTIC THERAPY

- Empiric use of antibiotics is recommended for patients who progress to SARS or septic shock associated to COVID-19, as follows:
- Initial empiric treatment for cases with no hypoxemia documented:
  - Oseltamivir 75mg *per oris* (PO) or via NET bid, for 5 days. Wait for the result of the viral panel before interrupting the drug before the fifth day.
- Initial empiric treatment of cases with documented hypoxemia:
  - Oseltamivir as above;
  - Ceftriaxone 1g, bid, IV, for 7 days in association with azithromycin 500mg PO or NET once a day, for 5 days. If infection by methicillin-resistant *Staphylococcus aureus* (MRSA) is suspected, ceftaroline 600mg bid, IV, for 7 days (or another antibiotic with broader coverage for Gram-negative bacteria, in case of suspected multidrug-resistant bacteria) in association with azithromycin 500mg PO or NET once daily, for 5 days.

## HEMODYNAMIC SUPPORT

**Figure 5:**
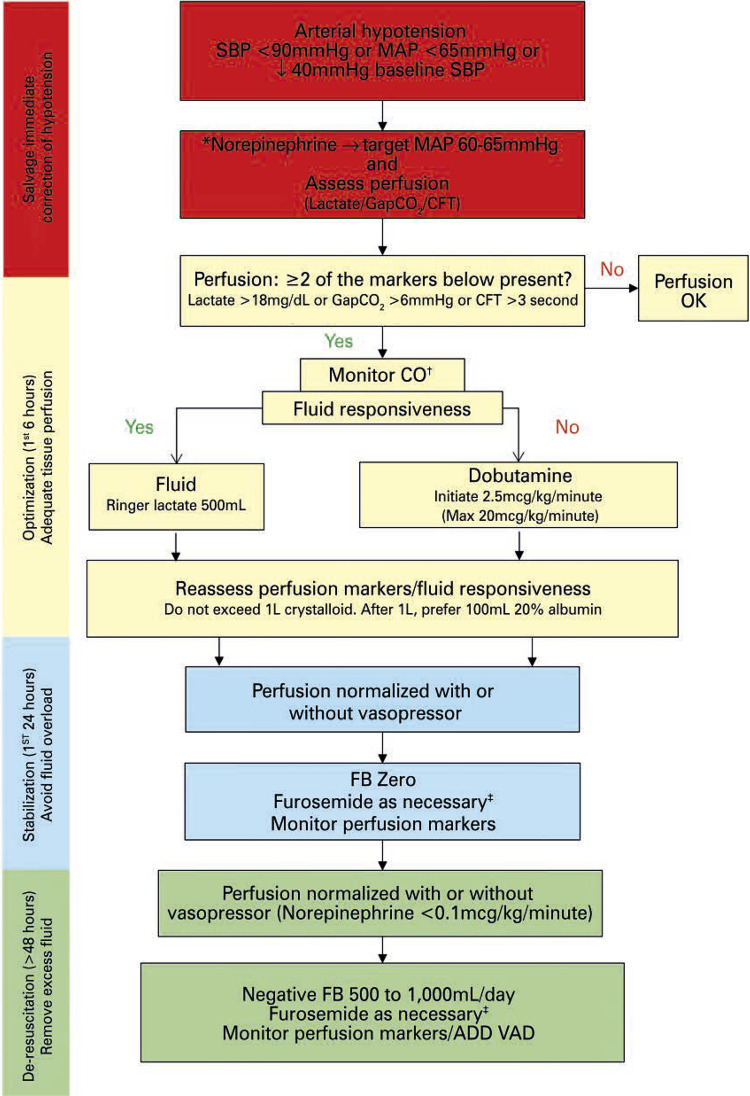
Algorithm for hemodynamic support of COVID-19 patients * Invasive monitoring of blood pressure and the insertion of a central venous catheter are suggested in patients receiving norepinephrine at a dose >0.1mcg/kg/minute and increasing. If norepinephrine dose >0.5mcg/kg/minute, we suggest starting epinephrine 0.01mcg/kg/minute. Start 200mg hydrocortisone continuous infusion if norepinephrine >0.2mcg/kg/minute after 6 hours of resuscitation; ^†^ monitor cardiac output if two or more perfusion parameters are abnormal; ^‡^ check fluid balance every 6 hours and adjust the diuretic dose according to the target (zero or negative fluid balance). Consider fluid balance as zero/positive in case of increase in vasopressor drugs, abnormal perfusion markers, laboratory signs of dehydration (hypernatremia or metabolic alkalosis), altered kidney function and non-measurable losses (fever and/or diarrhea). SBP: systolic blood pressure; MAP: mean arterial pressure; GapCO _2_ : difference between the partial pressure of venous carbon dioxide and the partial pressure of arterial carbon dioxide; CFT: capillary filling time; CO: cardiac output; FB: fluid balance; VAD: vasoactive drugs.

The algorithm for hemodynamic support of patients with COVID-19 is depicted in figure 5 .

- Invasive blood pressure (IBP) monitoring is suggested, as well as the insertion of a central venous catheter (CVC) in patients receiving norepinephrine at a dose >0.1mcg/kg/minute and increasing.
- Avoid using a peripherally inserted central catheter (PICC) to administer norepinephrine or maintain venous access, due to the increased risk of thrombotic events.
- If norepinephrine >0.5mcg/kg/minute, starting epinephrine 0.01mcg/kg/minute is suggested.
- The administration of hydrocortisone 200mg IV in CIP is recommended if norepinephrine >0.2mcg/kg/minute after 6 hours of resuscitation.

## PROPHYLAXIS

- Prophylaxis for stress ulcer: pantoprazole 40mg IV once a day.
- Prophylaxis for deep venous thrombosis (DVT):
  - Pneumatic compression (with no elastic stockings);
  - Initiate 5,000IU unfractionated heparin subcutaneous (SC), tid, for every patient, except when absolutely contraindicated.
  - D-dimer value will not change management for venous thromboembolism (VTE) prophylaxis with medication.

## ANTICOAGULATION

-  Do not use full anticoagulation based on the isolated D-dimer value.
-  Indicate full anticoagulation only when confirmed thromboembolic event or with formal indication – *e.g* . chronic atrial fibrillation.

## USE OF STEROIDS

-  If patient present septic shock, administer 200mg hydrocortisone IV via CIP, every 24 hours, as previously described ( Figure 5 ).
-  If patient progresses to need for MV and is not on hydrocortisone due to septic shock, initiate methylprednisolone 0.5mg/kg/day IV, for 7 days.
-  Corticosteroid withdrawal: begin after 7 full days of treatment. Reduction to 50% of the dose in D8 and to 25% of the dose in D9.

## GLYCEMIC CONTROL

-  Capillary blood glucose every 6 or 4 hours, according to the glycemic control protocol of the organization.
-  Avoid using insulin in CIP, to minimize exposure of the care team. Administer long-acting insulin early according to glycemic control of the previous 24 hours. However, if patient is receiving norepinephrine >0.2mcg/kg/minute, avoid administering SC insulin.

## SPECIFIC DRUGS FOR TREATING COVID-19

- Hydroxychloroquine: 400mg NET, bid, for 10 days is recommended. Do baseline and serial electrocardiogram (ECG) to check the QT interval. If corrected QT >500ms (or increment >60ms), re-evaluate risk-benefit, discontinue all additional drugs that prolong QT, including azithromycin. Maintain serum potassium levels >4.0mEq/L and magnesium >2.0mEq/L.
- Macrolides: azithromycin 500mg per day via NET, for 10 days, or clarithromycin 500mg IV, every 12 hours is recommended, if a patient is using a vasoactive drug. Especially in cases of concurrent use with hydroxychloroquine, ions and prolonged QT interval (corrected QT) should be monitored.
- Tocilizumab: its use is suggested only in selected cases, such as in patients with serum interleukin 6 (IL-6) level ten-fold above the upper normal limit or more, elevated D-dimer, thromboelastrometry suggestive of hypercoagulability state and sequential organ failure assessment (SOFA) score ^( 18 )^ ≥4, in addition to no presumed secondary bacterial infection, or liver dysfunction (international normalized ratio – INR >2.0 or total bilirubin – TB >2.0). Liver function should be monitored. The dose should be 4 to 8mg/kg (maximum unit dose: 800mg), with a maximum of three doses, every 12 hours.
- The use of lopinavir + ritonavir, remdesivir, convalescent serum, nitazoxanide, ivermectin and arbidol is restricted to clinical research protocols.

## ADDITIONAL TESTS

- The following laboratory tests should be sampled on daily basis during the ICU stay: complete blood count; kidney function (creatinine - Cr - and urea - Ur); electrolytes including sodium, potassium, magnesium, ionized calcium and phosphorus; arterial blood gases and lactate (if in hypoxemic MV and/or shock) and/or central venous blood gas (if with no IBP, or if weaning from MV, or not on MV and without hypoxemia and without shock) and C-reactive protein (CRP).
- If secondary bacterial infection is suspected, collect cultures and serial measurements of CRP and procalcitonin (PCT).
- Collect D-dimer every 48 hours (acts as a severity marker).
- Chest X-ray in case of clinical deterioration and always after procedures (OTI, CVC introduction, etc.).
- Consider daily ECG if the patient is using macrolides and hydroxychloroquine.
- Upon admission to ICU, collecting the daily tests described above, as well as troponin, type B natriuretic peptide (BNP), liver function, lactic dehydrogenase (DHL), ferritin, D-dimer, prothrombin time (PT), INR, activated partial thromboplastin time (aPTT), fibrinogen, thromboelastography, PCT, cultures, ECG and chest X-ray is recommended. Transthoracic echocardiography should be carried out within the first 24 hours of ICU admission.

## CONCLUSION

The clinical management of patients diagnosed with COVID-19 who develop severe forms of the disease and require intensive care is complex. Due to the complexity of patients, the exponential growth of new cases, and the high demand for supplies, human resources and trained professionals, critically-ill patients with COVID-19 are a major challenge for care teams and health systems. The evidence available in the literature for the treatment of patients with COVID-19 is scarce and limited to non-controlled studies. The recommendations presented in this document were developed to guide healthcare professionals who are directly involved in the care of COVID-19 patients, although predominantly based on experts’ opinions. Robust evidence from randomized controlled trials is required so that COVID-19 patients can be provided with more effective and safer treatments.
