# Supplementary material for: Intensive support recommendations for critically-ill patients with suspected or confirmed COVID-19 infection
Source: Einstein (Sao Paulo). 2020 May 29;18:eAE5793. doi: 10.31744/einstein_journal/2020AE5793 (PMC7259830; doi:10.31744/einstein_journal/2020AE5793)
Supplement: Translation

# Translation: Recomendações de suporte intensivo para pacientes graves com infecção suspeita ou confirmada pela COVID-19

**Authors:** Thiago Domingos Corrêa, Gustavo Faissol Janot de Matos, Bruno de Arruda Bravim, Ricardo Luiz Cordioli, Alejandra del Pilar Gallardo Garrido, Murillo Santucci Cesar de Assuncao, Carmen Silvia Valente Barbas, Karina Tavares Timenetsky, Roseny dos Reis Rodrigues, Hélio Penna Guimarães, Roberto Rabello, Frederico Polito Lomar, Farah Christina de La Cruz Scarin, Carla Luciana Batista, Adriano José Pereira, João Carlos de Campos Guerra, Bárbara Vieira Carneiro, Ricardo Kenji Nawa, Rodrigo Martins Brandão, Antônio Eduardo Pereira Pesaro, Moacyr Silva, Fabricio Rodrigues Torres de Carvalho, Cilene Saghabi de Medeiros Silva, Ana Claudia Ferraz de Almeida, Marcelo Franken, Marcele Liliane Pesavento, Raquel Afonso Caserta Eid, Leonardo José Rolim Ferraz

**Affiliations:** 1 Brasil Hospital Israelita Albert Einstein, São Paulo, SP, Brasil .

**Keywords:** Coronavírus, COVID-19, Insuficiência respiratória, Síndrome do desconforto respiratório do adulto, Unidades de terapia intensiva

## Abstract

Em dezembro de 2019, uma série de pacientes com pneumonia grave foi identificada em Wuhan, província de Hubei, na China. Esses pacientes evoluíram para síndrome respiratória aguda grave e síndrome do desconforto respiratório agudo. Posteriormente, a COVID-19 foi atribuída a um novo betacoronavírus, o coronavírus da síndrome respiratória aguda grave 2 (SARS-CoV-2). Cerca de 20% dos pacientes com diagnóstico de COVID-19 desenvolvem formas graves da doença, incluindo insuficiência respiratória aguda hipoxêmica, síndrome respiratória aguda grave, síndrome do desconforto respiratório agudo e insuficiência renal aguda e requerem admissão em unidade de terapia intensiva. Não há nenhum ensaio clínico randomizado controlado que avalie potenciais tratamentos para pacientes com infecção confirmada pela COVID-19 no momento da publicação destas recomendações de tratamento. Dessa forma, essas recomendações são baseadas predominantemente na opinião de especialistas (grau de recomendação de nível C).

## INTRODUÇÃO

Em dezembro de 2019, em Wuhan, província de Hubei, na China, uma série de pacientes evoluiu com pneumonia grave e apresentou síndrome respiratória aguda grave (SARS) e síndrome do desconforto respiratório agudo (SDRA).^( 1 , 2 )^Posteriormente, essa doença disseminou-se para outras regiões da China e diversos países em diferentes continentes, caracterizando uma pandemia.^( 3 )^Essa doença foi atribuída a um novo betacoronavírus, nomeado coronavírus da síndrome respiratória aguda grave 2 (SARS-CoV-2).^( 4 )^Recentemente, a doença causada por esse vírus recebeu o nome de *coronavirus disease 2019* (COVID-19).^( 5 )^

Os pacientes com COVID-19 apresentam, principalmente, febre, tosse, dispneia, mialgia e fadiga.^( 6 )^Embora a maioria deles apresente evolução favorável,^( 2 )^aproximadamente 20% dos pacientes desenvolvem formas graves da doença, incluindo insuficiência respiratória aguda hipoxêmica (IRpA), SARS, SDRA e insuficiência renal aguda (IRA) com necessidade de admissão em unidade de terapia intensiva (UTI).^( 1 , 2 , 7 )^Também já foi demonstrado que alguns grupos, especialmente os idosos e os portadores de outras patologias de base, têm maior risco de desenvolver disfunção de múltiplos órgãos (DMOS) e, por final, irem a óbito.^( 2 , 8 )^

No momento da publicação destas recomendações de tratamento, nenhum ensaio clínico randomizado e controlado (ECR) avaliava potenciais terapias para pacientes com infecção confirmada pela COVID-19.^( 5 , 9 )^Uma busca no ClinicalTrials.gov^( 10 )^utilizando o descritor “COVID-19” resultou em 179 ECR em fase de recrutamento de pacientes. Foi publicada recentemente uma revisão narrativa detalhada sobre as terapias específicas utilizadas para tratamento da COVID-19.^( 9 )^

Dessa forma, o material contido nestas recomendações é baseado predominantemente na opinião de especialistas. Assim, estas recomendações devem ser consideradas com cautela por profissionais de saúde, tendo em vista Grau de Recomendação de nível C (relato de casos, incluindo estudos de coorte ou caso-controle de menor qualidade).^( 11 )^

## CRITÉRIOS PARA ADMISSÃO NA UNIDADE DE TERAPIA INTENSIVA

É necessário apresentar pelo menos um dos critérios abaixo para internação em UTI:

- Pacientes com necessidade de oxigênio suplementar (cateter nasal de oxigênio – CNO_2_>3,0L/minuto) para manter saturação periférica de oxigênio (SpO_2_) >94% ou frequência respiratória (FR) ≤24rpm.
- Pacientes que necessitam de ventilação não invasiva (VNI) para manter SpO_2_>94% ou FR ≤24rpm.
- Insuficiência respiratória aguda com necessidade de ventilação mecânica (VM) invasiva.
- Pacientes com instabilidade hemodinâmica ou choque, definidos como hipotensão arterial (pressão arterial sistólica – PAS <90mmHg ou pressão arterial média – PAM <65mmHg), ou sinais de má perfusão orgânica ou periférica (alteração da consciência, oligúria, lactato ≥36mg/dL, entre outros), com ou sem utilização de vasopressores.
- Sepse com hipotensão arterial, necessidade de vasopressor ou lactato ≥36mg/dL.
- Choque séptico de acordo com os critérios Sepsis-3.^( 12 )^

## SUPORTE VENTILATÓRIO

Ventilação não invasiva e cateter nasal de alto fluxo:

- A utilização de VNI em modalidades de pressão positiva em vias aéreas em dois níveis de pressão (BiPAP) e de cateter nasal de alto fluxo (CNAF) está contraindicada pela grande produção de aerossol.
- Um teste curto de VNI (30 minutos) pode ser realizado para pacientes com IRpA. O teste de VNI deve ser realizado com parâmetros máximos de fração inspirada de oxigênio (FiO_2_) 50%, ou pressão positiva (PP) com delta 10cmH_2_O e pressão positiva expiratória na via aérea (EPAP) 10cmH_2_O ( Figura 1 ).
  **Figura 1:** Recomendações para utilização de ventilação não invasiva em pacientes com infecção suspeita ou confirmada pela COVID-19 * O teste de 30 minutos na VNI é realizado com máscara não ventilada (sem válvula exalatória) e circuito duplo em ventilador mecânico com filtro barreira na saída expiratória. IRpA: insuficiência respiratória aguda hipoxêmica; IOT: intubação orotraqueal; VNI: ventilação não invasiva; FR: frequência respiratória; SpO_2_: saturação periférica de oxigênio; FiO_2_: fração inspirada de oxigênio.
- Para permitir a utilização da interface de VNI com segurança, recomendamos a utilização de máscara orofacial não ventilada (sem válvula exalatória) acoplada a um circuito duplo, específico de VM, e conectado ao ventilador mecânico.
- Utilizar filtro barreira na saída expiratória dos ventiladores mecânicos.
- Critérios de sucesso na VNI: paciente tolerar interface e melhora do desconforto respiratório (FR <24rpm e SpO_2_≥94% com FiO_2_≤50%) após 30 minutos de VNI.
- Definição de dependência da VNI: necessidade de permanência em VNI superior ou igual a 4 horas em um período de 6 horas para manutenção da FR <24rpm e da SpO_2_≥94%.
- Pacientes dependentes da VNI devem ser transferidos para UTI precocemente, para realização de intubação orotraqueal (IOT) imediata.

## CRITÉRIOS PARA INTUBAÇÃO OROTRAQUEAL

- Pacientes que apresentam necessidade de oxigênio suplementar através de CNO_2_>5L/minuto ou VNI com FiO_2_>50% ou PP com delta >10cmH_2_O ou EPAP >10cmH_2_O para manter SpO_2_>94% ou FR ≤24rpm.
- Pacientes que não se adaptaram ou toleraram a interface de VNI.
- Pacientes dependentes de VNI.

## PROCEDIMENTOS PARA INTUBAÇÃO OROTRAQUEAL

- Paramentação da equipe durante o procedimento de IOT: avental impermeável, luva estéril, máscara N95, gorro, óculos de proteção e máscara protetora facial ( *face shield* ).
- Permanecer dentro do leito apenas os profissionais que participarão ativamente do procedimento. Recomendamos que um profissional capacitado permaneça na porta do quarto para eventual suporte durante a IOT.
- Preparar todo material para IOT, incluindo capnógrafo, drogas, fluidos e vasopressores, antes do início do procedimento e fora do leito.
- Vasopressor (noradrenalina) e cristaloides devem ser preparados e mantidos prontos para infusão antes do início do procedimento, pelo potencial risco de hipotensão arterial após a IOT.
- Utilizar cânula orotraqueal com aspiração subglótica e sistema de aspiração fechado em todos os pacientes submetidos à IOT e à VM.
- Todos os procedimentos de IOT devem ser realizados com videolaringoscopia direta.
- Todos os pacientes devem ser intubados com sequência rápida de intubação (SRI).
- É comum queda da SpO_2_abaixo de 70% imediatamente após a IOT. Portanto, a pré-oxigenação (O_2_100%) e o preparo adequado do paciente e dos materiais a serem utilizados para IOT são cruciais.
- Evitar ventilação com bolsa-válvula-máscara antes da IOT, pelo aumento de produção de aerossóis.
- Em pacientes sem utilização de VNI no momento da IOT: pré-oxigenação com máscara com reservatório com o menor fluxo de ar possível, para manter oxigenação efetiva. Evitar ventilação assistida com o dispositivo de bolsa-válvula-máscara ou uso de dispositivos supraglóticos, pelo potencial de aerossolização e contaminação dos profissionais.
- Em pacientes em uso VNI no momento da IOT: iniciar SRI com o paciente em VNI. Apenas retirar a VNI para realizar a intubação, ou seja, não retirar a máscara antes da IOT.
- Sequência das medicações para realização da SRI:
  - Fentanil 50 a 100mcg, endovenoso (EV).
  - Etomidato 0,3mg/kg EV ou propofol 2mg/kg EV, no mínimo 2 minutos após infusão do fentanil.
  - Lidocaína 2% sem vasoconstritor 40mg EV.
  - Bloqueio neuromuscular com succinilcolina 1,0mg/kg EV (ou rocurônio 1,2mg/kg EV, caso exista contraindicação à succinilcolina), para facilitar a IOT e evitar tosse durante o procedimento.
- Após verificação do adequado posicionamento do tubo orotraqueal com capnógrafo e instalação do filtro trocador de calor e umidade (filtro *heat and moisture* *exchanger –* HME) entre o tubo e o capnógrafo, o paciente pode ser conectado ao ventilador, com a colocação de filtro barreira *high efficiency particulate* *arrestance* (HEPA) na saída expiratória do ventilador para o ambiente.

## AJUSTES INICIAIS DO VENTILADOR MECÂNICO

Recomendamos os seguintes parâmetros iniciais de VM imediatamente após a IOT:

- Modo pressão controlada (PCV).
- Volume corrente (VC) de 6mL/kg de peso predito.^( 13 )^
- Pressão positiva expiratória final (PEEP) inicial de 15cmH_2_O.
- Frequência respiratória de 20 a 24rpm, para manter volume-minuto (VM) entre 7 e 10L/minuto.
- Pressão de distensão ( *driving pressure* ; pressão de platô menos PEEP) 15cmH_2_O.
- Alvo inicial de SpO_2_entre 92 e 96%.
- Alvo inicial de dióxido de carbono de final de expiração (EtCO_2_) entre 30 e 45.
- Sugerimos realização de gasometria arterial 1 hora após IOT, para eventuais ajustes nos parâmetros iniciais de VM.

## ESTRATÉGIA DE VENTILAÇÃO MECÂNICA PROTETORA

A estratégia de VM empregada em pacientes com diagnóstico suspeito ou confirmado de COVID-19 encontra-se detalhada na figura 2 .

**Figura 2:** Fluxograma para ventilação mecânica em pacientes com diagnóstico suspeito ou confirmado de COVID-19 * Se aumento da pressão parcial de CO_2_arterial e pH <7,20, avaliar possibilidade de reduzir a PEEP em 2cmH_2_O e reavaliar;^†^minititulação da PEEP com os parâmetros PEEP 25cmH_2_O e delta de pressão 15cmH_2_O durante 5 minutos. Otimizar sedação e BNM;^‡^seguir protocolo de VM prona. Caso não responda, realizar minititulação da PEEP novamente. Ajustar PEEP em 18cmH_2_O. IOT: intubação orotraqueal; VM: ventilação mecânica; PaO_2_/FiO_2_: relação entre pressão arterial parcial de oxigênio e fração inspirada de oxigênio; PEEP: pressão positiva expiratória final; CO_2_: gás carbônico; SpO_2_: saturação periférica de oxigênio; VM prona: ventilação mecânica em posição prona; PCV: modo pressão controlada; VC: volume corrente; FR: frequência respiratória; BNM: bloqueador neuromuscular.

## CRITÉRIOS PARA INÍCIO DE DESMAME VENTILATÓRIO

- Após 24 horas estável com PEEP incialmente ajustada conforme figura 2 , tentar reduzir PEEP de 1 em 1cmH_2_O, a cada 8 horas, para relação entre pressão arterial parcial de oxigênio e fração inspirada de oxigênio (PaO_2_/FiO_2_) >300.
- Se paciente for hipoxêmico crônico, utilizar PaO_2_/FiO_2_>250 para reduzir PEEP.
- Modificar o modo ventilatório controlado para espontâneo apenas quando PEEP ≤15cmH_2_O, FiO_2_<50% e escala de agitação e sedação de Richmond (RASS) >-5.^( 14 )^

## CRITÉRIOS PARA INÍCIO DE TESTE DE RESPIRAÇÃO ESPONTÂNEA

Para realização do teste de respiração espontânea (TRE), recomenda-se (figuras 3 and 4):

**Figura 3:** Fluxograma para desmame da ventilação mecânica em pacientes com diagnóstico suspeito ou confirmado de COVID-19 VM: ventilação mecânica; TRE: teste de respiração espontânea; VNI: ventilação não invasiva; CNO_2_: cateter nasal de oxigênio; *Bag* 100%: máscara de oxigênio com reservatório (não reinalante); PS: pressão de suporte; PEEP: pressão positiva expiratória final; FiO_2_: fração inspirada de oxigênio; SpO_2_: saturação periférica de oxigênio; PaCO_2_: pressão parcial de dióxido de carbono; FC: frequência cardíaca; PA: pressão arterial; FR: frequência respiratória.

**Figura 4:** Fluxograma de cuidados após teste de respiração espontânea e extubação de pacientes com diagnóstico suspeito ou confirmado de COVID-19 * Se paciente apresentar contraindicação para uso de VNI, manter em *bag* 100% e observar frequentemente. Manter VNI por 18 horas seguidas pós-extubação. Após esse período, caso o paciente não consiga permanecer 2 horas fora da VNI, deve-se realizar a Re-IOT. TRE: teste de respiração espontânea; IOT: intubação orotraqueal; EV: via endovenosa; VAS: via aérea superior; *Bag* 100%: máscara de oxigênio com reservatório (não reinalante); VNI: ventilação não invasiva.

- Permanecer por 24 horas em modo ventilatório por pressão de suporte (PSV) com PEEP=10cmH_2_O, FiO_2_<40% e pressão de suporte (PS) ≤10cmH_2_O e manter a pressão de oclusão das vias aéreas durante os primeiros 100 milissegundos (P0,1) ≤4cmH_2_O.
- Apresentar nível de consciência adequado: RASS 0-2^( 14 )^ou próximo ao basal.
- Apresentar estabilidade hemodinâmica, definida pela não necessidade de vasopressores ou necessidade de dose de noradrenalina <0,2mcg/kg/minuto ou dose estável de dobutamina ou em desmame e presença de marcadores de perfusão tecidual adequados.
- Após as 24 horas com os parâmetros de VM acima descritos e adequada troca gasosa, caracterizada por pH 7,3 com pressão arterial de dióxido de carbono (PaCO_2_) <55mmHg; PaO_2_≥60mmHg com FiO_2_≤40%; PEEP ≤10cmH_2_O; PaO_2_/FiO_2_≥250; SpO_2_≥90%, iniciar TRE (Figuras 3 e 4).
- Não realizar teste de escape do balonete ( *cuff leak test* )^( 15 )^antes de extubar os pacientes devido ao risco de aerossolização.

## ANALGESIA E SEDAÇÃO

- A associação entre propofol (dose máxima de 3,0mg/kg/hora) EV e fentanil (dose de 25 a 50mcg/hora; dose máxima 100mcg/hora) EV é a primeira escolha para sedação e analgesia de pacientes submetidos à VM por COVID-19.
- Na presença de propofol em dose superior a 3mg/kg/hora, recomenda-se associar midazolam, na dose de 0,02 a 0,2mg/kg/hora EV.
- Alvo de sedação recomendado durante as primeiras 48 horas de VM: RASS -5.
- Alvo de sedação recomendado após as primeiras 48 horas de VM:
  - Relação PaO_2_/FiO_2_>250 e PEEP ≤15cmH_2_O e FiO_2_≤50%: RASS -3 a zero.
  - Relação PaO_2_/FiO_2_≤250 e/ou PEEP ≥15cmH_2_O: RASS -4 a -5.
- Caso seja necessário, realizar doses adicionais para controle de agitação durante a VM:
  - Propofol: 10 a 40mg EV em bólus.
  - Midazolam: 3 a 5mg EV em bólus.
  - Para controle adicional da dor (analgesia) durante a VM, recomendamos doses adicionais de fentanil em bólus (50mcg EV) e associação com analgésicos comuns (exemplo: dipirona ou paracetamol) com objetivo de poupar a utilização de opioides.
- Em relação à utilização de bloqueador neuromuscular (BNM), recomenda-se administração de cisatracúrio 0,15mg/kg EV em bólus, seguida de infusão em bomba de infusão contínua (BIC) de 1 a 4mcg/kg/minuto, quando houver:
  - Assincronia grave persistente, caracterizada por piora da oxigenação e da ventilação, após ajuste da VM e de sedação, em pacientes com PaO_2_/FiO_2_entre 150 e 200;
  - PaO_2_/FiO_2_<150 com PEEP >15cmH_2_O.
  Em pacientes recebendo BNM, com base nas diretrizes vigentes,^( 16 , 17 )^recomenda-se:
    - Índice bispectral (BIS) com alvo de 40 a 60, com taxa de supressão de surtos (SR) > zero.
    - *Train of four* (TOF): alvo zero (avaliar uma vez por dia, se possível).
    - Na impossibilidade de monitorização com BIS, deve-se garantir sedação profunda (RASS-5) antes do início da infusão do BNM.
- Sugere-se manutenção de BNM por 48 horas após seu início em pacientes com COVID-19. Pacientes em uso de BNM por mais de 48 horas, se apresentarem PaO_2_/FiO_2_>200 e PEEP <20cmH_2_O, retirar BNM e reavaliar em 24 horas.
- Em pacientes recebendo sedação contínua, recomenda-se rever diariamente a possibilidade de diminuição da sedação, se pH >7,30 e:
- Se PaO_2_/FiO_2_>200, tentar trocar midazolam por propofol, caso esteja em uso de midazolam.
- Se PaO_2_/FiO_2_>300, tentar trocar propofol por dexmedetomedina (0,3 a 1,3mcg/kg/hora).
- Tentar, concomitantemente, reduzir progressivamente a dose de fentanil administrada.
- Se durante tentativa de diminuição da sedação, o paciente apresentar assincronia com queda da SpO_2_e/ou instabilidade hemodinâmica, sugere-se:
  - Administrar 10 a 40mg de propofol EV em bólus e 25 a 50mcg de fentanil EV em bólus.
  - Administrar uma dose de neurolépitco (Quetiapina 25 a 50mg via sonda nasoenteral – SNE – ou, se não puder via SNE, utilizar haloperidol EV).
  - Se não houver melhora após 20 minutos, aprofundar a sedação, diminuindo em -2 na RASS, ou até RASS -5, e reavaliar em 24 horas. Se necessários dois ou mais bólus em um período de 6 horas, considerar aprofundar a sedação e reavaliar em 24 horas.
- Após 48 horas de sedação, em pacientes em uso crônico de drogas psicoativas, realizar reconciliação medicamentosa, antes da considerar reduzir sedação contínua.

## SUPORTE CLÍNICO GERAL

- Higienização das mãos sempre. Instituir precauções de contato e aerossol durante toda permanência na UTI.
- Evitar drogas que alargam o intervalo QT (exemplo: zofran, bromoprida, fluconazol, metoclopramida antiarrítmicos), sobretudo se em uso de antibióticos macrolídeos e cloroquina.
- Não realizar inaloterpia, objetivando-se evitar aerossolização. Utilizar broncodilatadores com espaçador em caso de broncoespasmo.

## ANTIBIOTICOTERAPIA

- Recomenda-se a administração de antibióticos empíricos para pacientes que evoluem para SARS ou choque séptico associado à COVID-19 como se segue:
- Tratamento empírico inicial de casos sem hipoxemia documentada:
  - Oseltamivir 75mg via oral (VO) ou via SNE a cada 12 horas, por 5 dias. Aguardar resultado do painel viral para suspensão da medicação antes do quinto dia.
- Tratamento empírico inicial de casos com hipoxemia documentada:
  - Oseltamivir conforme orientação acima;
  - Ceftriaxone 1g, a cada 12 horas EV, por 7 dias em associação à azitromicina 500mg VO ou SNE uma vez ao dia, por 5 dias. Na suspeita de infecção por *Staphylococcus* *aureus* resistente à meticilina (MRSA), ceftarolina 600mg a cada 12 horas EV por 7 dias (ou outro antibiótico com cobertura mais ampla para bactérias *Gram* -negativas, em caso de suspeita de bactérias multirresistentes) em associação com azitromicina 500mg VO ou SNE uma vez por dia, por 5 dias.

## SUPORTE HEMODINÂMICO

O algoritmo para suporte hemodinâmico de pacientes com COVID 19 está apresentado na figura 5 .

**Figura 5:** Algoritmo para suporte hemodinâmico de pacientes com COVID-19 * Sugerem-se a monitorização da pressão arterial invasiva e a inserção de cateter venoso central em pacientes recebendo noradrenalina em dose >0,1mcg/kg/minuto e em ascensão. Se dose de noradrenalina >0,5mcg/kg/minuto, sugerimos iniciar adrenalina 0,01mcg/kg/minuto. Iniciar hidrocortisona 200mg em infusão contínua, se noradrenalina >0,2mcg/kg/minuto ao final de 6 horas de ressuscitação;^†^monitorar débito cardíaco se dois ou mais parâmetros de perfusão estiverem alterados;^‡^verificar balanço hídrico a cada 6 horas e ajustar dose de diurético conforme a meta (balanço hídrico zerado ou negativo). Considerar balanço hídrico zerado/positivo em caso de aumento de vasopressor, alteração dos marcadores de perfusão, sinais laboratoriais de desidratação (hipernatremia ou alcalose metabólica), alteração da função renal e perdas não mensuráveis (febre e/ou diarreia). PAS: pressão arterial sistólica; PAM: pressão arterial média; GapCO_2_: diferença entre a pressão parcial de dióxido de carbono venoso e a pressão parcial de dióxido de carbono arterial; TEC: tempo de enchimento capilar; DC: débito cardíaco; BH: balanço hídrico; DVA: drogas vasoativas.

- Sugerem-se a monitorização da pressão arterial invasiva (PAI) e a inserção de cateter venoso central (CVC) em pacientes recebendo noradrenalina em dose >0,1mcg/kg/minuto e em ascensão.
- Evitar utilização de cateter central de inserção periférica (PICC) para administração de noradrenalina ou manutenção de acesso venoso, devido ao risco aumentado de eventos trombóticos.
- Se dose de noradrenalina >0,5mcg/kg/minuto, sugere-se iniciar adrenalina 0,01mcg/kg/minuto.
- Recomenda-se a administração de hidrocortisona 200mg EV em BIC, se noradrenalina >0,2mcg/kg/minuto ao final de 6 horas de ressuscitação.

## PROFILAXIAS

- Profilaxia para úlcera de estresse: pantoprazol 40mg EV uma vez ao dia.
- Profilaxia para trombose venosa profunda (TVP):
  - Compressor pneumático (sem meias elásticas);
  - Iniciar heparina não fracionada, na dose de 5.000UI por via subcutânea (SC), a cada 8 horas para todos os pacientes, salvo contraindicações absolutas.
- O valor do dímero D não alterará a conduta em relação à profilaxia medicamentosa para tromboembolismo venoso (TEV).

## ANTICOAGULAÇÃO

- Não instituir anticoagulação plena, com base no valor isolado do dímero D.
- Indicar anticoagulação plena apenas se evento tromboembólico confirmado ou nos casos em que exista indicação formal – por exemplo, fibrilação atrial crônica.

## UTILIZAÇÃO DE CORTICOIDE

- Se paciente apresentar choque séptico, administrar hidrocortisona 200mg EV em BIC, a cada 24 horas, conforme descrito anteriormente ( Figura 5 ).
- Se paciente evoluir para necessidade de VM e não estiver em uso de hidrocortisona devido a choque séptico, iniciar metilprednisolona 0,5mg/kg/dia EV, por 7 dias.
- Retirada do corticoide: início após 7 dias completos de tratamento. Redução para 50% da dose no D8 e para 25% da dose no D9.

## CONTROLE GLICÊMICO

- Realizar glicemia capilar a cada 6 ou 4 horas, conforme protocolo de controle glicêmico institucional.
- Evitar utilização de insulina em BIC, com o objetivo de minimizar exposição da equipe assistencial. Administrar insulina de longa duração precocemente conforme controle glicêmico das 24 horas anteriores. Entretanto, se paciente com noradrenalina >0,2mcg/kg/minuto, evitar administrar insulina SC.

## DROGAS ESPECÍFICAS PARA TRATAMENTO DA COVID-19

- Hidroxicloroquina: recomenda-se a utilização na dose de 400mg SNE a cada 12 horas por 10 dias. Realizar eletrocardiograma (ECG) basal e seriado para controle do intervalo QT. Se QT corrigido >500ms (ou incremento >60ms), reavaliar risco-benefício, suspender todos os fármacos adicionais que alargam QT, incluindo a azitromicina. Manter níveis séricos de potássio >4,0mEq/L e de magnésio >2,0mEq/L.
- Macrolídeos: recomenda-se utilização de azitromicina 500mg por dia via SNE por 10 dias ou claritromicina 500mg EV, a cada 12 horas, se paciente em uso de droga vasoativa. Notadamente, nos casos de uso concomitante à hidroxicloroquina, recomenda-se monitorar íons e alargamento do intervalo QT (QT corrigido).
- Tocilizumabe: sugere-se sua utilização apenas em casos selecionados, como de pacientes com nível sérico de interleucina 6 (IL-6) dez vezes acima do limite superior da normalidade ou mais, dímero D elevado, tromboelastrometria sugestiva de hipercoagulabildade e escore *sequential organ failure* *assessment* (SOFA)^( 18 )^≥4, além de ausência de infecção bacteriana secundária presumível, e de disfunção hepática (razão normalizada internacional – RNI >2,0 ou bilirrubina total – BT >2,0). A função hepática deve ser monitorada. A dose deve ser de 4 a 8mg/kg (dose máxima unitária: 800mg), sendo no máximo três doses, com intervalo de 12 horas entre elas.
- A utilização de lopinavir + ritonavir, remdesivir, soro convalescente, nitazoxanida, ivermectina e arbidol está restrita a protocolos de pesquisa clínica.

## EXAMES SUBSIDIÁRIOS

- Recomenda-se considerar a coleta diária durante a permanência na UTI dos seguintes exames laboratoriais: hemograma completo; função renal (creatinina – Cr – e ureia – Ur); eletrólitos incluindo sódio, potássio, magnésio, cálcio iônico e fósforo; gasometria arterial e lactato (se em VM hipoxêmica e/ou choque) e/ou gasometria venosa central (se sem PAI, ou se em desmame de VM, ou fora da VM e sem hipoxemia e sem choque) e proteína C-reativa (PCR).
- Se suspeita de infecção bacteriana secundária, colher culturas e seriar os biomarcadores PCR e procalcitonina (PCT).
- Coletar dímero D a cada 48 horas (serve como marcador de gravidade).
- Realizar radiografia de tórax se piora clínica e sempre após procedimentos (IOT, passagem de CVC etc.).
- Considerar ECG diário se paciente em uso de antibióticos macrolídeos e hidroxicloroquina.
- Recomenda-se, no momento da admissão na UTI, a coleta dos exames diários descritos anteriormente e troponina, peptídeo natriurético do tipo B (BNP), função hepática, desidrogenase lática (DHL), ferritina, dímero D, tempo de protrombina (TP), RNI, tempo de tromboplastina parcial ativada (TPPa), fibrinogênio, tromboelastograma, PCT culturas, ECG e radiografia de tórax. Realizar ecocardiograma transtorácico nas primeiras 24 horas de admissão na UTI.

## CONCLUSÃO

O manejo clínico de pacientes com diagnóstico de COVID-19 que desenvolvem formas graves da doença e necessitam de cuidados intensivos é complexo. Devido à complexidade dos pacientes, ao crescimento exponencial de novos casos, e à alta demanda de insumos, recursos humanos e profissionais capacitados, pacientes graves com COVID-19 representam um grande desafio para equipes assistenciais e os sistemas de saúde. As evidências disponíveis na literatura para tratamento de pacientes com COVID-19 são escassas e limitadas a estudos não controlados. As recomendações apresentadas neste documento foram desenvolvidas para direcionar os profissionais de saúde que estão envolvidos diretamente na assistência de pacientes com COVID-19, embora elaboradas predominantemente a partir da opinião de especialistas. Evidências robustas provenientes de ensaios clínicos randomizados e controlados são necessárias para que possamos oferecer tratamentos mais efetivos e seguros para pacientes com COVID-19.
